# The orthodontist’s reach in bullying

**DOI:** 10.1590/2177-6709.24.2.015-016.edt

**Published:** 2019

**Authors:** Flavia Artese

**Affiliations:** 1 Universidade do Estado do Rio de Janeiro, Departamento de Odontologia Preventiva e Comunitária (Rio de Janeiro/RJ, Brazil).

Even though contemporary orthodontics has broadened the age span for orthodontic treatment in adults, teenagers will always constitute a major portion of our patients. After all, as it is well known, orthodontic treatment by itself can only provide dramatic dentofacial changes during pubertal growth spurt, which may benefit our patients both in function and esthetics, and in the so-called psychosocial aspect.

It is exactly about the association between orthodontic treatment need, oral health-related quality of life (OHRQoL), and bullying that the original article by Gatto et al, published in this issue of the DPJO, looked at, in a sample of 815 Brazilian teenagers. Interestingly, they found that there was an association between OHRQoL and bullying. For example, teenagers with negative bullying consequences were three times more prone to have low scores for OHRQoL. A similar correlation was also observed in Jordanian children in a study performed by Al-Omari et al,^1^ in 2014. They found a significant relationship between bullying due to dentofacial features and negative effects on OHRQoL. Indeed, teeth have been reported to be the most frequently targeted feature for bullying, followed by physical strength and body weight. The most common dental features targeted by bullies were spacing between teeth, missing teeth, shape and color of teeth, and prominent upper incisors.^2^

However, when looking closely at this information, I questioned myself on how much, as a caregiver, do I really know about bullying. As an orthodontist, I understand the standard of care for treating malocclusions, but since I see so many teenagers, do I really know the underlying consequences of bullying? Can I truly sense that my patient is a bullying victim due to his/her malocclusion? And if I don’t and I wait longer for him/her to be treated, what are the possible consequences in the long run?

Bullying is described as a specific form of aggression with an imbalance of power, whereby a more powerful individual repeatedly and intentionally causes harm to a weaker individual.^3^ There are different ways of bullying, which can be direct or indirect. Direct bullying includes physical and verbal acts of aggression, such as hitting or name-calling; while indirect bullying is described as social exclusion and rumor spreading.^4^ Prevalence rates can range between 10% and 60% of teenagers, with a variation of 100 million to 600 million adolescents directly involved in it each year in the world.^3^

It is believed that bullying cannot be considered as a simple conduct disorder,^4^ and it has been proposed to be a result of impoverished individual or environmental factors and the ensuing maladaptive development.^3^ Still, strange though it may be, it is nowadays considered by psychologists as an evolutionary adaptation with the purpose of improving somatic resources, mate selection, assertion of dominance and social status.^3^ This might explain why bullying is found in different cultures and geographic regions, why it is not limited to modern civilization, and why so many teenagers are involved in it.

Involvement in bullying is not so straightforward as it is generally believed to be an aggressor/victim relationship. There are distinct groups which can be divided into bullies, victims, and a third group called bullies/victims. Bullies are characterized as being aggressive, hostile, domineering, and exhibit little anxiety or insecurity. On the other hand, victims in general are more depressed, anxious and insecure, with a lower level of self-esteem and a more internalizing behavior. They are usually lonelier at school, have few good friends and have an escape behavior, such as avoiding going to school or other public places. The third group, which was unknown to me, the bullies/victims group, are those who both bully and are victimized. Not much information is available for this group, but they seem to also have externalizing and aggressive behavior, have lower academic skills and social acceptance.^5^

The long-term effects of bullying were very well summarized in a recent review paper focusing on controlled prospective studies of pre-existing health conditions, family situation, and other exposures to violence.^4^ Victims followed from childhood to adulthood had an increased risk for internalizing problems, anxiety disorder, and depression, as well as reports of poorer general health, more body pain and slow recovery from illnesses. Also, victims were found to have lower educational qualifications, be worse on financial management and earn less than their peers. Bully/victims had a slighter higher risk for anxiety, depression, psychotic experiences, and suicide attempts than pure victims. Not much is known on pure bullies, but they seem to have no increased risk for mental or health issues, but are more likely to be less educated, unemployed or even involved in crime.

So, in summary, bullying is quite a frequent behavior, very common in adolescents, with psychological and health long term effects on victims and/or on bullies, and malocclusion can be an important target for bullying. It becomes quite clear to me that we, as orthodontists, should deepen our knowledge about this problem. For example, adequate approaches should be created to address this issue in our first consultation with the parents and/or the child, and if bullying is reported, psychological support should be referred. After all, our reach as orthodontists in this matter seems to require extreme responsibility. We may be the first health care professionals to have access to bullying reports, since it is so intimately associated with our specialty.^1^ ^,^ ^2^ Quite some food for thought in our everyday practice...

Good readings.
